# CMV serostatus and T-cell repertoire diversity 5 years after allogeneic hematopoietic stem cell transplantation

**DOI:** 10.1038/s41375-023-01836-w

**Published:** 2023-02-16

**Authors:** Zuleika Calderin Sollet, Antonia Schäfer, Sylvie Ferrari-Lacraz, Stavroula Masouridi-Levrat, Anne-Claire Mamez, Amandine Pradier, Federico Simonetta, Yves Chalandon, Jean Villard, Stéphane Buhler

**Affiliations:** 1 Transplantation Immunology Unit and National Reference Laboratory for Histocompatibility, Department of Diagnostic, Geneva University Hospitals, Geneva, Switzerland; 2 Service of Haematology, Department of Oncology, Geneva University Hospitals and Faculty of Medicine, University of Geneva, Geneva, Switzerland

**Keywords:** Bone marrow transplantation, Transplant immunology, Genetics

## To the Editor

Allogeneic hematopoietic stem cell transplantation (HSCT) is an effective therapy for hematologic malignancies. During hematopoiesis reconstitution, the dynamic interactions between the graft’s immune cells and the recipient’s tissues lead to alloreactivity, which can be associated with the positive graft-versus-tumor effect but can also lead to a severe complication like graft-versus-host disease (GVHD).

Early after HSCT, T-cell homeostasis in the recipient is controlled by the peripheral expansion of alloreactive and pathogen-specific donor memory T cells or residual recipient cells [1]. Later, the reconstitution proceeds by de novo production of naïve T cells from donor precursors migrating into the thymus from the recipient’s bone marrow [2, 3]. CD8^+^ T cells require up to 1 year to reach physiological values [4, 5], while CD4^+^ T cells take 2 years or even longer, depending on patients’ conditions, such as GVHD, to achieve standard counts and full function [6].

**Fig. 1:**
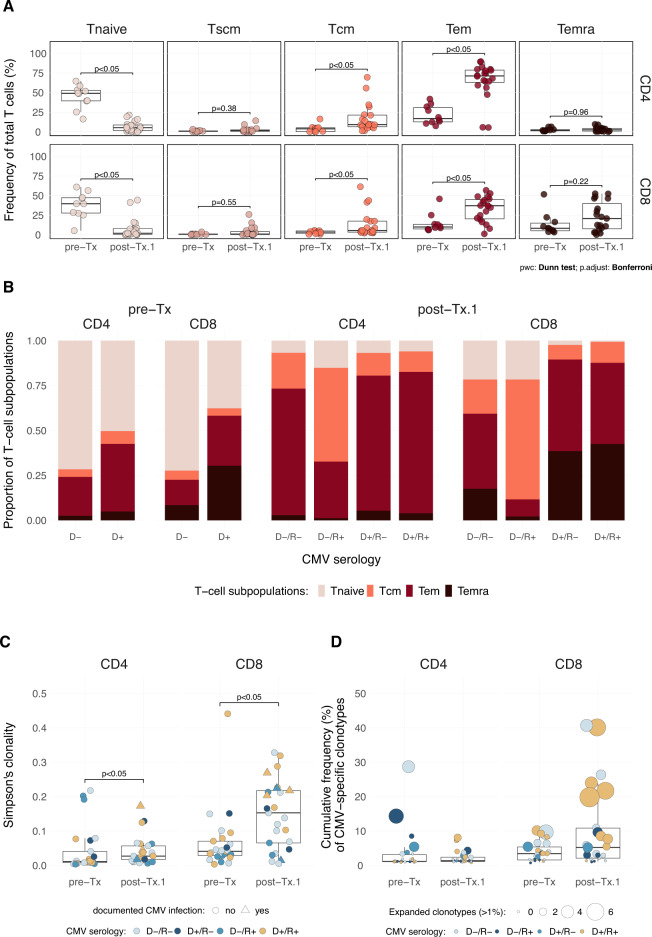
Overview of the CD4^+^ and CD8^+^ T-cell phenotypes and TCR diversity from *n* = 25 1-year post-HSCT patients (post-Tx.1) and their donors (pre-Tx). **A** Frequency of the T_naive_, T_scm_, T_cm_, T_em_, and T_emra_ cells in the CD4^+^ and CD8^+^ T-cell populations. Box plots show the median frequency per group, and dots correspond to one subject. **B** Proportion of T_naive_, T_cm_, T_em_, and T_emra_ subsets within four sets of donor/recipient pairs (D/R) classified on their CMV serotype (D-/R-, D+/R-, D-/R+ and D+/R+). Only the donor status is reported for pre-Tx data. Each bar plot displays the four T cell subsets distribution in CD4^+^ and CD8^+^ compartments at both time points. **C** Simpson’s clonality of the TCR CDR3β repertoire from CD4^+^ and CD8^+^ fractions. Each dot corresponds to a subject and is color-coded according to the CMV serologic status (see B). **D** Clonal expansion of CMV-specific clonotypes. Box plots show the cumulative frequency of CMV-specific clonotypes. The dot’s size denotes the number of the most abundant CMV-specific clonotypes (i.e., expanded clones with a frequency above 1%). D: donor, R: recipient, (−) CMV negative, (+) CMV positive. Boxes show median and interquartile ranges, and dots correspond to individuals. Dunn’s test was used for multiple comparisons between groups for each T cell fraction. *P* values below 0.05 were considered statistically significant.

Using high throughput sequencing of the TCR beta complementarity-determining region 3 (CDR3), we reported the TCR repertoire in a cohort of 116 patients 1-year post-HSCT. Compared to the donor’s repertoire before transplantation, we observed a more oligoclonal profile that depends on the patient’s and donor’s age [7]. Moreover, we demonstrated that the cytomegalovirus (CMV) serologic status and CMV infection/reactivation post-HSCT are the most significant clinical features influencing the reconstitution of the repertoire. In this study, we had the opportunity to analyze 26 patients of the previous cohort 5 to 6 years after HSCT. The first study was performed on the bulk of T cells; therefore, we aimed to explore the diversity of TCR repertoire according to a subset of CD4^+^ and CD8^+^ T-cell populations. For this part, T-cell differentiation was assessed in 25 other patients who received HSCT from related and unrelated donors (Supplementary Table 1). To evaluate CD4^+^ and CD8^+^ T-cell differentiation, we determined the frequency of naïve (T_naive_), stem-memory (T_scm_), central memory (T_cm_), effector memory (T_em_), and RA-expressing memory (T_emra_) T cells in pre-Tx and post-Tx.1 (i.e., at 1 year) samples. In post-Tx samples, we observed a reduced proportion (<5%) of naïve T cells in both populations (Fig. 1A). In contrast, T_cm_ and, more noticeably, T_em_ and CD8^+^ T_emra_ cells reached significantly higher frequencies than before HSCT. In the CD8^+^ compartment (Fig. 1B), we observed a drastic decrease in T_naive_ cells and an increased proportion of T_em_ and T_emra_ cells in the D+/R- and D+/R+ groups. A less marked but still noticeable decrease of T_naive_ cells was also observed in the D-/R- and D-/R+ groups. Regarding the CD4^+^ compartment, T_emra_ cells only represented a small portion of the repertoire at both time points. Globally, the most notable change post-Tx was in the D-/R+ group with an increased frequency of T_cm_ cells (Fig. 1B).

The repertoire diversity (Fig. 1C) switched from polyclonal values in donors to more oligoclonal profile in recipients at 1 year, with the CD8^+^ repertoire showing the most extreme reduction of diversity. Furthermore, the change in clonality was more evident in CMV-positive samples, particularly in the donor/recipient positive pairs. We observed an increased cumulative frequency and number of CMV-specific clonotypes in the CD8^+^ compartment post-HSCT (Fig. 1D), better highlighted in the D+/R+ pairs.

**Fig. 2:**
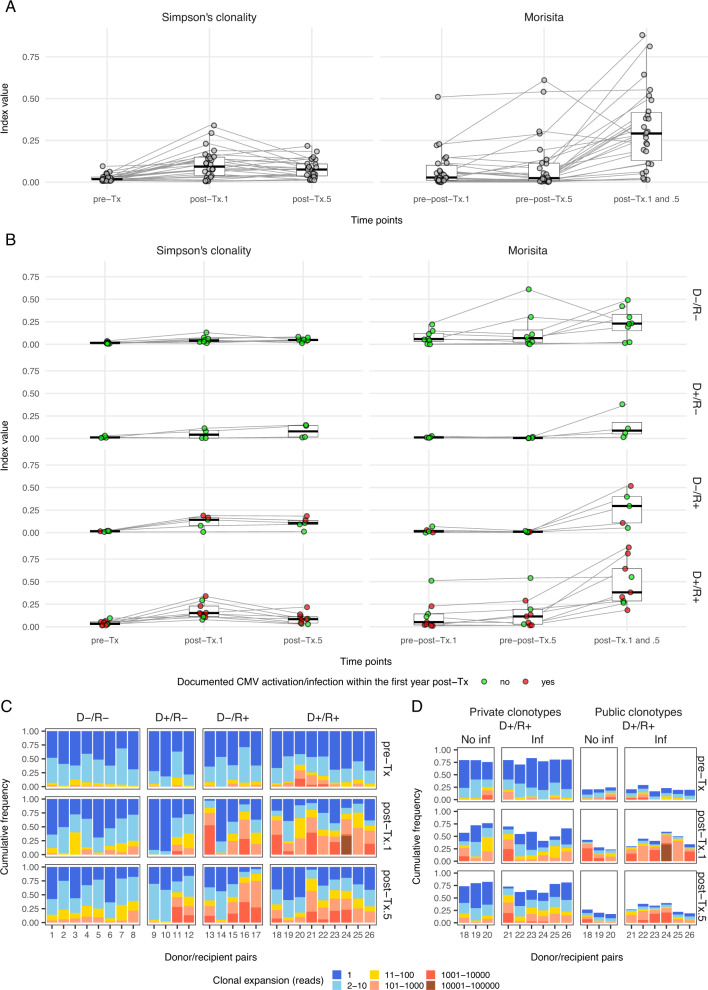
Overview of the diversity and overlap of the TCR CDR3β repertoire in 26 donor/recipient pairs as measured by Simpson’s clonality and Morisita’s index, respectively. **A** Three time points were considered and compare the repertoire in 10 related and 16 unrelated donors prior to transplantation (pre-Tx), at 1 year post-HSCT in 26 full chimeric recipients (post-Tx.1) and their follow-up at 5 or 6 years (post-Tx.5). At the latter time point, 23 recipients were still fully chimeric, while 3 had mixed chimerism (i.e., recipient chimerism estimated between 7% and 15% in peripheral blood mononuclear cells according to an analysis performed with microsatellite markers). Clonality and Morisita’s values are shown along the y axis with lines connecting donors and recipients. In **B**, Simpson’s clonality and Morisita’s index are plotted according to CMV serologic status and a documented CMV reactivation or infection (i.e., defined as CMV DNA in plasma above the limit of detection, currently 2.1E + 1 UI/ml, in patients with or without clinical symptoms) within the first year post-transplantation. Of note, no CMV reactivation or infection was documented in the last year prior to the follow-up at 5 or 6 years. The clonal expansion of the repertoire across the three time points is presented in **C** according to CMV serologic status. The cumulative frequency of clonotypes for a given category is plotted with color representing the level of expansion considered based on the number of sequenced reads. **D** provides a detailed view of the expansion of the repertoire in the nine D+/R+ pairs. The clonotypes are categorized as “private” (i.e., observed in only one donor/recipient pair of the current cohort) or “public” (i.e., observed in two or more donor/recipient pairs) and the data are stratified according to occurrence of CMV reactivation or infection within the first year post-transplantation. D: donor CMV negative (−) or positive (+), R: recipient CMV negative (−) or positive (+), Inf: CMV reactivation or infection within the first year post transplantation, Tx: allogeneic HSCT.

In the second part, we evaluated the long-term evolution of the TCR repertoire in 26 patients from our original cohort of 116 patients [7] at 5–6 years post-HSCT. The diversity was significantly reduced in 1-year post-HSCT samples (post-Tx.1) (*p* = 1.3e-6). However, the repertoire’s diversity remained quite stable when comparing 5–6 years post-HSCT samples (post-Tx.5) to post-Tx.1 samples (*p* = 0.18, Fig. 2A, left). This was confirmed in the D-/R-, D+/R- and D-/R+ groups as the clonality did not change over time (Fig. 2B left). Conversely, in D+/R+ pairs exhibiting the most drastic shift of clonality at 1 year, the repertoire showed an opposite trend with the recovery of slightly more diverse profiles. While the overlap of clonotypes remained very low in recipients at 5–6 years compared to their donor’s profile, a greater overlap was observed between the two post-HSCT time points (Fig. 2A, right), especially in D+/R+ pairs (Fig. 2B, right). The degree of clonal expansion increased in the post-Tx.1 group and, to a lesser extent, in post-Tx.5 compared to pre-Tx, mainly when the recipient was positive for CMV (Fig. 2C). Most expanded clonotypes post-HSCT in D+/R+ pairs were public (Fig. 2D). Interestingly, a decrease in the cumulative frequency of expanded clonotypes occurred between post-Tx.1 and post-Tx.5 in D+/R+, especially when there was no documented CMV infection/reactivation (Fig. 2D).

Although the TCR repertoire changed drastically regarding clonal diversity within the first year post-HCST, it evolved to a more stable profile throughout the follow-up of 5–6 years. Interestingly, a large TCR overlap was observed between 1 and 5 years in patients with a documented CMV infection/reactivation within the first year. CMV has been shown to drive the expansion of virus-specific memory T cells that accumulate over time, a process called T-cell memory inflation [8]. Remarkably, in the D+/R+ group, CMV infection/reactivation seemed to induce a T cell response that involves highly expanded public TCR clones, notably at 1-year post-HSCT.

Our previous publication did not assess the TCR diversity according to CD4^+^ or CD8^+^ subpopulations. These data were generated in an independent cohort, indicating that the TCR diversity is more pronounced in the CD4^+^ T cell compartment. A peripheral expansion of memory CD4^+^ and CD8^+^ T cells at the expense of naïve T cells during the first year post-HSCT was observed. It could be due to a reduced naïve pool linked to a reduced thymic output or the lymphopenic environment at the periphery [9]. Patients at risk for CMV reactivation were almost deprived of CD8^+^ T_naive_ cells, similar to a previous report [10]. The CD8^+^ T cells exhibited the most skewed TCR profile, which might be driven by T_em_ or T_emra_ subsets [10].

In summary, the diversity of the TCR repertoire post-HSCT established at 1 year remains stable over time. Furthermore, the TCR repertoire diversity is reduced in the CD8^+^ compartment, compared to the CD4^+^, mainly influenced by the CMV status.

## Supplementary information

Supplemental material

## Data Availability

The datasets generated during and/or analyzed during the current study are available in the Yareta repository, 10.26037/yareta:ue7dlyulbzelzoqbek34g47ovm.
