# Supplementary material for: CMV serostatus and T-cell repertoire diversity 5 years after allogeneic hematopoietic stem cell transplantation
Source: Leukemia. 2023 Feb 16;37(4):948–51. doi: 10.1038/s41375-023-01836-w (PMC10079542; doi:10.1038/s41375-023-01836-w)
Supplement: Supplementary file 1 — Supplemental material [file 41375_2023_1836_MOESM1_ESM.docx]

**Supplementary information**

**Materials/Subjects and Methods**

*Patient and donor samples*

This study includes patients who had received an allogeneic HSC graft at Geneva University Hospitals in Switzerland and showed no sign of relapse.

An independent cohort of 25 pairs of patients at one-year post-HSCT (post-Tx.1) and donors as controls pre-HSCT (pre-Tx) (Table S1) was used for analyses on CD4^+^ and CD8^+^ fractions. Then, 26 donor/recipient pairs (1) with a follow-up at five to six years post-HSCT (post-Tx.5) was analyzed longitudinally. Whole blood samples from patients were collected at the time mentioned above and freshly processed at the Swiss National Reference Laboratory for Histocompatibility (LNRH). In addition, the donor’s peripheral blood mononuclear cells (PBMCs) were cryo-preserved until analysis.

*CD4 and CD8 positive T-cell isolation*

CD4^+^ and CD8^+^ T cells were isolated using the MACS technology (Miltenyi Biotech, Gmbh). In brief, whole blood samples were incubated for 15 minutes at 4⁰C with the CD4 or CD8 magnetic beads (StraightFrom Whole Blood CD4 or CD8 MicroBeads, Miltenyi Biotech, Gmbh). Then, labeled samples were loaded onto a MACS column on a MACS separator and further eluted as the positively selected cell fraction. In parallel, donor-matched CD4^+^ and CD8^+^ fractions were obtained from cryopreserved PBMCs by negative selection with magnetic beads (CD4^+^ or CD8^+^ T cell Isolation Kit, Miltenyi Biotec, GmbH).

*FACS analysis*

The purity of CD4^+^ and CD8^+^ fractions was determined by flow cytometry using CD3-PE-Vio770, CD4-PE, and CD8-VioBright-FITC antibodies (Miltenyi Biotech, Gmbh). We considered samples with less than 10% contamination between both T-cell populations for the study.

T-cell differentiation was assessed using a panel of recombinant antibodies composed of CD4- or CD8-FITC, CD45RO-PE, CCR7-PerCP-Vio700, and CD95-APC (Miltenyi Biotech, Gmbh). So, the naïve (T_naive_, CD45RO^-^CCR7^+^CD95^-^), stem memory (T_scm_, CD45RO^-^CCR7^+^CD95^+^), central memory (T_cm_, CD45RO^+^CCR7^+^CD95^+^), effector memory (T_em_, CD45RO^+^CCR7^-^CD95^+^) or TEM cells re-expressing CD45RA (T_emra_, CD45RO^-^CCR7^-^CD95^+^) were identified. Cellular immunostaining was carried out following the manufacturing instructions. Each subset was referred to as a percent of the specified cell population. Data were acquired in BD Accuri™ C6 Plus Flow Cytometer and analyzed with FlowJo software (FlowJo_v10.8.1, Beckman Coulter).

*DNA extraction*

Genomic DNA (gDNA) was extracted using the Genomic DNA extraction kit NucleoSpin (Machery-Nagel, Düren, Germany). Alternatively, gDNA was automatically purified with the EZ1 Advanced XL machine (QIAGEN, GmbH).

*TCR Immunosequencing*

Up to 30ng/ul of genomic DNA was used to prepare the TCR library with the ImmunoSEQ kit (Adaptive Biotechnologies, Seattle, WA). Then, high throughput sequencing of the TCR CDR3β region was carried out at survey resolution on the Illumina HiSeq system (HiSeq Reagent 150-cycle V3 kit, Illumina, San Diego, USA) following a multiplex PCR (ImmunoSEQ© assay, Adaptive Biotechnologies, Seattle, USA). Raw sequenced data were transferred into the ImmunoSEQ analyzer application for clonotype quantitation and annotation. Further, productive TCR CDR3β rearrangements were retrieved from the Adaptive platform to estimate their number and frequency along with diversity and differential abundance metrics.

*Statistical analyses*

# TCR CDR3β clonotype’s frequencies were calculated from the number of sequenced templates and used to describe the evenness amongst a set of T cells. In addition, the TCR repertoire overlap before and after HSCT was estimated by Morisita’s index (2). This index is based on CDR3 sequences similarities and ranges from 0 (no overlap) to 1 (complete overlap). Besides, Simpson’s clonality metric was used to estimate the TCR repertoire diversity as it is less sensitive to variances in sample size than Shannon’s clonality. Clonality expands from 0 to 1, defining maximum diversity (polyclonal) at the lowest indices and minimal diversity (oligoclonal) at values near 1. Clonotypes were considered public if retrieved in more than one recipient/donor pair from the cohorts.

# Pairwise comparisons between groups were performed with a paired Wilcoxon exact signed-rank test, while a Dunn’s test was used for multiple comparisons between groups. P-values below 0.05 were considered statistically significant. All the analyses were performed in R version 4.0.3 (2020-10-10) using different packages, including ggplot2, reshape2, dplyr, rstatix.

*Identification of CMV-specific clonotypes*

Clonotypes were considered specific to CMV if their CDR3β sequence was identical to one of the sequences of a set of CMV-reactive clonotypes retrieved from the McPAS-TCR(3) and VDJdb (4) databases. In addition, to capture more CMV- specific clonotypes in our cohort, we used a generalized Levenshtein distance (adist, R Utils package 3.6.2) where a cost of one was given to those clonotypes having only one amino acid substitution. Then the absolute number of CMV-specific clonotypes found at more than 1% was calculated for each cell compartment and transplant group.

Table S1. Patient characteristics

| Characteristic | Donor/Recipient pairs (n = 25) |
| --- | --- |
| Age of patients | Median=54.72, Min= 17.85, Max=73.41 |
| Sex | Male = 15, Female=10 |
| Donor type | HLA-identical sibling = 4  HLA-matched unrelated = 14  HLA-mismatched unrelated = 4  HLA-mismatched relative ≥ 2MM = 3 |
| Primary disease | Acute Myeloid Leukemia = 10,  Myelodysplastic syndrome = 7,  Chronic myelomonocytic leukemia = 1,  Acute lymphoblastic leukemia = 1,  Chronic lymphocytic leukemia = 1,  Chronic myeloid leukemia = 1,  Lymphoma = 3  Myeloma = 1 |
| Conditioning | Myeloablative = 6 (ATG = 4, TCD = 1, ATG-TCD = 1)  Reduced intensity = 19 (ATG = 13, ATG-TCD = 2, no ATG = 4)  ATG (Anti-Thymocyte Globulin), TCD (T Cell Depletion) |
| GVHD grade | No GVHD = 5, grade 1 = 12, grade ≥2 = 8 |
| Donor Lymphocyte Infusion (number) | 18 (n=0), 5 (n=1), 1 (n=2), 2 (n=3) |
| Donor/Recipient (D/R) CMV serotype | D-/R- = 10, D+/R- = 2, D-/R+ = 5, D+/R+ = 8, |

**References**

1. Buhler S, Bettens F, Dantin CA, Ferrari-Lacraz S, Ansari Djaberi MG, Mamez A-C, et al. Genetic T-cell receptor diversity at 1 year following allogeneic hematopoietic stem cell transplantation. Leukemia. 2022;34(5):1422-32.

2. Rempala GA, Seweryn M. Methods for diversity and overlap analysis in T-cell receptor populations. Journal of mathematical biology. 2013;67(6-7):1339-68.

3. McPAS-TCR: A manually-curated catalogue of pathology-associated T cell receptor sequences [Internet]. 2017 [cited August 2021].

4. Goncharov M, Bagaev D, Shcherbinin D, Zvyagin I, Bolotin D, Thomas PG, et al. VDJdb in the pandemic era: a compendium of T cell receptors specific for SARS-CoV-2. Nat Methods. 2022.
